# Genomic resources for a model in adaptation and speciation research: characterization of the *Poecilia mexicana* transcriptome

**DOI:** 10.1186/1471-2164-13-652

**Published:** 2012-11-21

**Authors:** Joanna L Kelley, Courtney N Passow, Martin Plath, Lenin Arias Rodriguez, Muh-Ching Yee, Michael Tobler

**Affiliations:** 1 Department of Genetics, Stanford University, 300 Pasteur Dr, Stanford, CA, 94305, USA; 2 Department of Zoology, Oklahoma State University, 501 Life Sciences West, Stillwater, OK, 74078, USA; 3 J.W. Goethe University Frankfurt/M., Biologicum, Evolutionary Ecology Group, Max-von-Laue Str. 13, 60438, Frankfurt am Main, Germany; 4 División Académica de Ciencias Biológicas, Universidad Juárez Autónoma de Tabasco (UJAT), C.P. 86150, Villahermosa, Tabasco, Mexico

**Keywords:** *De novo* assembly, Ecological speciation, Expression analysis, Poeciliidae, RNA sequencing, Transcriptome

## Abstract

**Background:**

Elucidating the genomic basis of adaptation and speciation is a major challenge in natural systems with large quantities of environmental and phenotypic data, mostly because of the scarcity of genomic resources for non-model organisms. The Atlantic molly (*Poecilia mexicana*, Poeciliidae) is a small livebearing fish that has been extensively studied for evolutionary ecology research, particularly because this species has repeatedly colonized extreme environments in the form of caves and toxic hydrogen sulfide containing springs. In such extreme environments, populations show strong patterns of adaptive trait divergence and the emergence of reproductive isolation. Here, we used RNA-sequencing to assemble and annotate the first transcriptome of *P. mexicana* to facilitate ecological genomics studies in the future and aid the identification of genes underlying adaptation and speciation in the system.

**Description:**

We provide the first annotated reference transcriptome of *P. mexicana*. Our transcriptome shows high congruence with other published fish transcriptomes, including that of the guppy, medaka, zebrafish, and stickleback. Transcriptome annotation uncovered the presence of candidate genes relevant in the study of adaptation to extreme environments. We describe general and oxidative stress response genes as well as genes involved in pathways induced by hypoxia or involved in sulfide metabolism. To facilitate future comparative analyses, we also conducted quantitative comparisons between *P. mexicana* from different river drainages. 106,524 single nucleotide polymorphisms were detected in our dataset, including potential markers that are putatively fixed across drainages. Furthermore, specimens from different drainages exhibited some consistent differences in gene regulation.

**Conclusions:**

Our study provides a valuable genomic resource to study the molecular underpinnings of adaptation to extreme environments in replicated sulfide spring and cave environments. In addition, this study adds to the increasing number of genomic resources in the family Poeciliidae, which are widely used in comparative analyses of behavior, ecology, evolution, and medical genetics.

## Background

A fundamental challenge in evolutionary biology is the mechanistic integration from genomic variation to fitness of organisms in their natural environment. Linking genomes to fitness is requisite to understand adaptation, speciation, and the interactions between the two processes [1,2]. In the past, different model systems have been used to either understand the genomic basis of phenotypes, or the fitness effects of phenotypic variation in response to different environmental conditions in nature, but in only few systems have we a thorough understanding about the ecological context in which phenotypic traits evolve and the genomic basis of respective traits. Notable exceptions include heavy metal tolerance in *Arabidopsis*[3], eco-morphological differentiation in lake trout [4], whitefish [5,6], and marine snails [7], the reduction of armor in freshwater sticklebacks [8,9], and changes in fur coloration in beach mice [10].

Elucidating the genomic basis of adaptation and speciation in systems with in- depth knowledge of ecological sources of selection and phenotypic trait variation has been hindered by the lack of genomic resources, which are often only available for model organisms. However, next generation sequencing (NGS) techniques provide a promising tool in this endeavor [11-13]. While whole genome sequencing on replicated sets of individuals is still expensive, focusing on transcribed portions of the genome (the transcriptome) has become increasingly popular. Such transcriptomic studies focus on sequencing cDNA libraries constructed from mRNA isolated from specific tissues (RNA-seq [14,15]), and in combination with barcoding technologies [16,17], can provide large amounts of sequence and expression level data for comparative transcriptomic studies.

We provide a first characterization of the transcriptome of the Atlantic molly, *Poecilia mexicana* (Poeciliidae). This livebearing fish species is widely distributed in freshwater environments along the Atlantic versant from northeastern Mexico into lower Central America [18,19]. *Poecilia mexicana* has been used as a study organism in animal behavior and behavioral ecology [20-22], predator–prey interactions [23,24], sensory ecology [25-27], and life history evolution [28,29]. Furthermore, the species is the maternal ancestor of a unisexual hybrid species, the Amazon molly (*P. formosa*), and is thus frequently investigated to address questions about the evolution and maintenance of sexual reproduction [30-32].

Most importantly, *P. mexicana* is an emerging model system to study adaptation to extreme environments and ecological speciation, as the species has colonized both hydrogen sulfide-rich and cave habitats in southern Mexico [33]. Hydrogen sulfide (H_2_S) is a potent toxicant lethal for most metazoans even in micro-molar amounts by inhibiting cellular respiration [34,35]. The absence of light in caves inhibits the use of visual senses, and cave-dwellers must cope with perpetual darkness, especially if they evolved from diurnal surface-dwelling forms like in poeciliids [36,37]. Extreme habitats harbor distinct ecotypes of *P. mexicana* that have evolved convergently across independently colonized sulfidic springs and caves [38,39]. Compared to conspecifics in adjacent non-sulfidic surface habitats, which harbor the ancestral populations, extremophile populations diverged in eye size, body shape, and gill morphology [38,40], physiology [38], life history strategies [41,42], and behavior [43,44]. Despite the lack of physical barriers between extreme and adjacent normal habitats, gene flow across habitat types is eminently low [45], and reproductive isolation is at least partially mediated by natural and sexual selection against immigrants [46-48].

Despite the well-characterized selective environments and phenotypic variation in this system, there are currently no genomic resources to start addressing questions about the genomic changes underlying trait divergence. Such resources are particularly required to test whether fixed positively selected mutations and changes in gene expression patterns show similar patterns of convergence across replicated environmental gradients, or whether unique genomic changes in each evolutionary replicate essentially precipitated similar phenotypic effects. To address such questions in the future, and to start building genomic resources for other study areas using *P. mexicana* (and close relatives such as the sailfin molly *P. latipinna*, the amazon molly *P. formosa*, and the guppy *P. reticulata*), we used RNA sequencing to obtain a first transcriptome of this species. We particularly focused on gill tissues, since many physiological processes involved in the maintenance of homeostasis take place here [49,50], and used six individuals from ancestral, non-sulfidic populations to facilitate *de novo* transcriptome assembly in absence of a reference genome. Our key objectives were to (1) create a database of the gill transcriptome in *P. mexicana* for future comparative studies, (2) to annotate transcripts based on functional annotations in reference databases, (3) to compare the *P. mexicana* transcriptome to other published fish transcriptomes, including the guppy (*Poecilia reticulata*), medaka (*Oryzias latipes*), threespined stickleback (*Gasterosteus aculeatus*), and zebrafish (*Danio rerio*), and (4) to identify potential candidate genes of interest in the study of extremophile poeciliids.

## Construction and content

### Sample collection methods

Gill samples for transcriptomic profiling were obtained from fish collected in their natural environment. Three adult females were collected in both the Arroyo Rosita (Río Pichucalco drainage) and Arroyo Bonita (Río Tacotalpa drainage). Both river drainages originate in the mountains of the Sierra Madre de Chiapas of Southern Mexico and flow into the wide floodplains of northern Tabasco, where they ultimately join the Río Grijalva (see Additional file 1: Figure S1 for a map). Collection locations were intermediate gradient, non-sulfidic streams with similar structure and water chemistry (see [38] for detailed environmental data). Locations were chosen to represent the most eastern (Tacotalpa) and most western (Pichucalco) drainages inhabited by sulfide spring fishes in Southern Mexico [38]. Analyses were restricted to specimens from habitats with similar environmental conditions (i.e., non-sulfidic surface streams) to facilitate *de novo* assembly.

Fish were caught with a seine (2 × 6 m). Immediately after capture, fish were sacrificed, measured and weighed, and gill tissue was extracted from both sides of the body using previously sterilized scissors and forceps. Tissues were preserved in 2 ml of RNAlater (Ambion, Inc.) and stored on ice during transport to the laboratory. Experiments were approved by the Institutional Animal Care and Use Committee of Oklahoma State University (ACUP AS10-15).

### RNA isolation and RNAseq library construction

RNA was isolated from gills by pulverizing 50–100 mg of tissue frozen in liquid nitrogen in individual tubes with a Covaris Cryoprep at setting 3. RNA was then extracted with Qiagen’s RNeasy Plus mini kit. PolyA+ mRNA was prepared from 50 μg total RNA using Invitrogen’s Dynabeads mRNA purification kit. RNA was bound and eluted twice to Dynabeads to minimize ribosomal RNA contamination. mRNA was fragmented to an average size of 400 nt using NEB’s mRNA Fragmentation Module by incubation at 94°C for 4 minutes. Fragmented mRNA was purified using Agencourt RNAClean XP beads and eluted in 12 μl ddH_2_O. First strand cDNA was synthesized in a 20 μl reaction using Invitrogen’s Double-stranded cDNA kit, primed with 1 μl of a mix of random hexamers:oligo dT primers (2 μg:1 μg), and incubated with Superscript II at 45°C for one hour. The first strand cDNA reaction was used directly in NEBNext mRNA Second Strand Synthesis kit.

After second strand cDNA synthesis, the reaction was purified with Agencourt Ampure XP beads and eluted in 25 μl water. Double stranded cDNA was used as input for Illumina sequencing library preparation with end-repair using the NEBNext end-repair kit, A-tailing with Taq polymerase, ligation with Truseq barcoded adapters, and amplification with Kapa Library Amplification Readymix. All steps were cleaned up with Ampure XP beads. RNAseq libraries were quantified on an Agilent 2100 Bioanalyzer High Sensitivity DNA chip and pooled based on nM concentration. Libraries were sequenced on an Illumina HiSeq 2000 with paired-end 101 bp reads.

### De novo assembly

Reads were sorted by barcode and trimmed to 87 basepairs (5 bases were trimmed from the beginning of each read and 11 bases from the end of the read due to lower sequence quality at the beginning and end). All reads with remaining ambiguous bases were removed and only paired reads were used for the analysis, which resulted in removal of less than 5% of reads. Data from the six individuals was concatenated for *de novo* assembly using Trinity [51], with the default settings. To remove possible spurious transcripts and very lowly expressed transcripts, a modified version of RSEM (RNA-Seq by Expectation Maximization [52]) available with the Trinity package was applied, and transcripts with an FPKM (fragments per kilobase of exon per million fragments mapped) less than 0.1 were removed. To remove isoforms and paralogs, we conducted a reciprocal blast to our own dataset. For sequences with >97% similarity, we only retained the longest sequence for further analysis. Finally, we tested for predicted open reading frames (ORFs) using OrfPredictor [53]. Only sequences with a predicted ORF were retained for subsequent analyses. Six transcripts were validated using Invitrogen SuperScript One-Step reverse-transcriptase-polymerase chain reaction (RT-PCR) with primers designed for each transcript (Additional file 2: Table S1). Nested internal primers were used in a second PCR step with Kapa Library Amplification Readymix polymerase (Kapa Biosystems), the product of which was Sanger sequenced for validation.

### Comparison to other transcriptomes

We compared the reduced dataset to the NCBI Unigene records (ftp://ftp.ncbi.nih.gov/repository/UniGene/) for medaka (*Oryzias latipes*; 21,803 transcripts), three-spined stickleback (*Gasterosteus aculeatus*; 18,681 transcripts), and zebrafish (*Danio rerio*; 52,653 transcripts)*.* All datasets were downloaded on 11/1/2011. We also compared our data set to the guppy (*Poecilia reticulata*; 71,138 transcripts) transcriptome [54], which was obtained from http://www.bio.fsu.edu/kahughes/Databases.html. Reciprocal similarity searches were conducted using tblastx with an E-value threshold of 0.001.

### Transcriptome annotation

To annotate transcripts, we first conducted a blast search of all unique contigs with a predicted ORF against the SwissProt database (http://ca.expasy.org/sprot/; blastx, critical E-value = 0.001; database accessed 11/04/2011) using Blast2GO [55-57]. Any sequences that did not have a match in SwissProt were subsequently blasted against the NCBI non-redundant (NR) protein database (blastx, critical E-value = 0.001; database accessed 12/03/2011). This procedure was employed because the SwissProt database provides more informative functional annotations, but the NR database is larger and has the possibility to annotate sequences not available in SwissProt. For each sequence, we retained the top blast hit for subsequent analysis. Lastly, contigs with no match in either database were translated and searched against the Pfam-A and Pfam-B protein families databases [58] with an E-value cut-off of 0.01, and against the Rfam database for non-coding RNA families [59].

Sequences with a match in either the SwissProt or NR database were subsequently annotated with Gene Ontology (GO) IDs [60] as implemented in Blast2GO. GO IDs describe gene product characteristics and are hierarchically organized in terms of biological processes, molecular functions, and cellular components. Due to the hierarchical organization, GO annotations can be simplified to a smaller set of high-level GO terms (GO slims). We obtained GO slims through Blast2GO with the generic slim developed by the GO Consortium (http://www.geneontology.org/GO.slims.shtml).

To compare transcriptome annotation in *P. mexicana* to previously published annotations of *P. reticulata*[54], the reduced *P. reticulata* dataset (http://www.bio.fsu.edu/kahughes/Databases.html) was re-annotated with the same procedure as outlined above for *P. mexicana* to reduce effects of differential methodologies and database access dates. Differential representation of records in each GO slim term was then compared between the two species by counting the number of sequences associated with each GO slim category. We tested for differences in representation for each GO slim category with a Chi-square test.

Finally, we searched the *P. mexicana* transcriptome for candidate genes of interest in future comparative studies. We particularly focused on genes related to environmental stress and living in extreme environments. To that end, we searched our annotation database for gene products known to be involved in general stress and oxidative stress responses. Since *P. mexicana* has also colonized several springs with high concentrations of hydrogen sulfide and severe hypoxia, we also searched for gene products related to sulfide detoxification and metabolism as well as hypoxia induced responses.

### SNP discovery

To facilitate future research on genomic variation in *P. mexicana*, we developed a database of single nucleotide polymorphisms (SNPs). Putative SNPs were identified by mapping trimmed RNAseq reads to the reference transcriptome using Burrows-Wheeler Aligner (BWA [61]). We then applied the Genome Analysis Toolkit (GATK [62]) to the mapped reads for PCR duplicate removal, base quality score recalibration, and indel realignment. SNP and INDEL discovery as well as genotyping was performed across all 6 samples using standard hard filtering parameters [63]. The resulting SNPs were annotated as synonymous or non-synonymous using an in-house script based on the open reading frame predicted for each transcript. We also quantified the number of fixed alternate alleles between *P. mexicana* individuals from the two drainages.

### Differential expression analysis

We tested whether there are differences in gene expression patterns in the three biological replicates of *P. mexicana* from the two different drainages. Trimmed reads were mapped to the reference transcriptome using Bowtie [64] and RSEM [52] within the Trinity package suite [51]. Mapped read counts were highly correlated between individuals (among and within drainages; Pearson correlation: *r* ≥ 0.91 for log-transformed data and *r* ≥ 0.99 for un-transformed data in all cases). We then used the edgeR package of Bioconductor [65-67] to identify genes that are differentially expressed between the drainages. We used the common dispersion estimated from a negative binomial implemented in edgeR. To reduce bias in our analyses due to low or high expression in single individuals, we filtered lowly expressed transcripts and those that were only expressed in a small number of samples by selectively retaining transcripts with at least one count per million in at least 3 samples. Of the 53,245 original transcripts, 21,480 meet these criteria. Sequences that were differentially expressed across drainages were annotated based on the procedure described above.

## Utility

### Transcriptome assembly

Sequencing gill tissue transcriptomes in six females of *P. mexicana* yielded over 70 million reads (Table 1), which represented – on average – 23.7-fold coverage of the transcriptome for each individual; taking the transcriptome size of 49.5 Mb, the sum of the number of bases in our assembled transcriptome, as a reference. We assembled reads, using Trinity, into 80,111 contigs. We excluded 14,982 contigs with an FPKM less than 0.1. After a reciprocal blast search within the *P. mexicana* transcriptome, we maintained the longest of those contigs presenting more than 97% similarity to each other. This filtering procedure allowed us to discard 11,884 smaller, redundant contigs and to retain 53,245 contigs representing putatively unique loci. Over 99% of these loci exhibited a predicted open reading frame (ORF), and only 331 sequences were removed from further analyses because they did not have an ORF. Data files with sequence information were deposited in the Sequence Read Archive on Genbank (Study Accession ID: SRP014728).

**Table 1:** **Sequencing and assembly statistics for Illumina sequencing used for the assembly of the *P. mexicana *transcriptome**

Average number of reads (± SD)	11,698,857 (2,363,343)
Total number of reads	70,193,146
Average number of base pairs (± SD)	1,181,584,624 (238,697,721)
Total number of base pairs	7,089,507,746
Average coverage (± SD)	23.8 (4.4)
Total number of assembled contigs	80,111
Total number of unique loci	53,245
Total number of unique loci with predicted ORF	52,914
Mean contig length (basepairs) (± SD)	932 (1004)
Maximum contig length	15,623

Data presented represents reads from *N* = 6 bar coded individuals.

**Figure 1:**
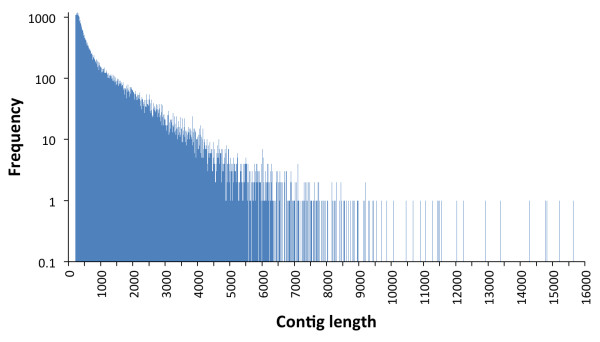
Frequency distribution of assembled contigs by size.

The frequency distribution of contig lengths is depicted in Figure 1. As expected, the number of mapped reads per contig was significantly correlated with contig length (Pearson correlation on log-transformed values: *r* = 0.87, *P* < 0.001). The average (± SE) level of gene expression controlled for contig length was 19.4 (± 1.2) FPKM, ranging from a minimum of 0.02 to a maximum of 33,072.50.

Six predicted transcripts were selected for RT-PCR and sequencing validation (Additional file 2: Table S1). All six predicted transcripts were validated via direct sequencing.

### Comparison to other fish transcriptomes

We compared the *P. mexicana* transcriptome to data available from four other freshwater fishes (guppy, medaka, stickleback, and zebrafish; Table 2) using reciprocal blast searches. Mapping unique *P. mexicana* contigs to the transcriptomes of these species resulted in blast matching of more than 50% (51-74%) in each species examined. In contrast, matching was lower (29-55%) when mapping contigs from those species to the *P. mexicana* dataset. The difference in the percent mapping between the species partially reflects the difference in the sizes of the databases and the phylogenetic divergence among species under consideration (Additional file 3: Figure S2).

**Table 2:** **Results of reciprocal blast searches of the *P. mexicana *transcriptome to the database of the guppy (*Poecilia reticulata*) **[54]**, and Uniprot databases of medaka (*Oryzias latipes*), zebrafish (*Danio rerio*), and stickleback (*Gasterosteus aculeatus*) **[68]

	***P. mexicana *to focal species**			**Focal species to *P. mexicana***		
	**Unique *P. mexicana *transcripts**	**Unique hits in focal species**	**% coverage in focal species**	**Unique focal species transcripts**	**Unique hits in *P. mexicana***	**% coverage in *P. mexicana***
Guppy	29,671	46,634	65.6	46,953	29,337	55.2
Medaka	19,447	15,337	70.3	14,958	18,277	34.4
Zebrafish	25,013	26,871	51.0	26,870	24,370	45.8
Stickleback	18,037	13,807	73.9	13,554	17,024	28.8

The table lists the number of unique transcripts that map to the reference species’ transcriptome, the number of unique transcripts that received hits in the reference species, and the percent coverage in the references species database.

### Annotation

The *P. mexicana* transcriptome was annotated by blast searches against the SwissProt and the NCBI non-redundant (NR) protein databases. Overall, 26,317 contigs (49.7%) had matches in the SwissProt database. These represented 17,814 unique records. Of the remaining sequences, 3,475 (6.6%) had a match to 2,497 unique records in the NR database. Of the unmatched contigs, 766 had matches in the Pfam database and 31 had matches in the Rfam database, indicating at least part of unmatched contigs represent real transcript.

**Figure 2:**
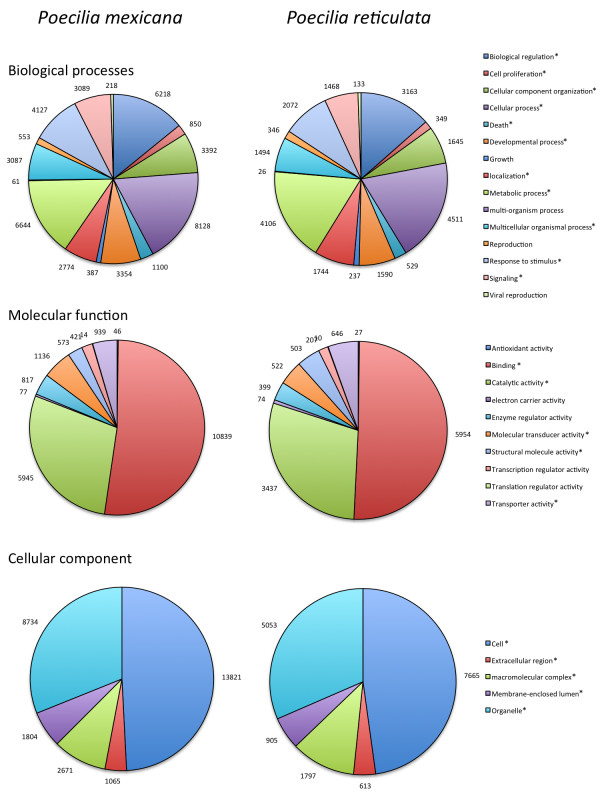
**Blast2GO assignment for 17,286 *P. mexicana *and 9,721 *P. reticulata *sequences. **Significant differences in the frequency of different categories are highlighted with an asterisk. The numbers next to each colored slice of the pie chart represent the number of genes in the respective category.

The 29,792 sequences with a match in the SwissProt or NR databases were further annotated with Gene Ontology (GO) terms based on the Uniprot database, which yielded results for 22,184 (74.5%) of sequences. Of these, 13,002 sequences were annotated with a biological process GO term, 13,623 with a molecular function, and 14,430 with a cellular component. The relative frequency of level 2 GO terms is visualized in Figure 2. We also compared the representation of records in each generic slim between *P. mexicana* and *P. reticulata*. While representation was qualitatively very similar between the two species, 20 out of 30 level 5 GO categories exhibited significant differences across species even when accounting for the effects for multiple testing (*Chi*^2^ ≥ 13.545, *P* ≤ 0.0002, *α*’ = 0.001).

By mining our annotation database, we found a diverse set of candidate genes for future genomic studies related to life in extreme environments (Table 3). The candidate list includes genes involved in general and oxidative stress responses. Particularly, we found multiple gene products associated with different functions from the heat shock protein family and could detect several components of antioxidant systems. Due to the severe hypoxia and high concentrations of hydrogen sulfide in some *P. mexicana* habitats, we also surveyed our data for genes previously associated with responses to hypoxia and sulfide detoxification. Most notably, we recorded a hypoxia specific transcription factor and several components involved in regulating aerobic and anaerobic metabolism. Furthermore, our database contains records for sulfide:quinone oxidoreductase, rhodanese, and several other key proteins involved in the metabolic processing of toxic hydrogen sulfide.

**Table 3:** Candidate genes with annotations from the SwissProt (SP) database that are involved in environmental stress tolerance, hypoxia, and sulfide metabolism

**Category**	**Gene description**	**Contig ID**	**Accession number**	**Percent coverage**	**E-value**	**Number of contigs**
General stress responses						
Stress-activated protein kinase signaling pathways [69]	Stress-activated protein kinase (JNK3)	comp49081_c0_seq1	P53779	97	2.71E-63	1
	Stress-activated protein kinase kinase (SAPKK4)	comp30986_c0_seq1	O14733	96	2.43E-153	1
Highly inducible heat shock proteins [70,71]	Hsp 70	comp24087_c0_seq1	P27541	92	3.65E-103	1
		comp34171_c0_seq1	Q91233	98	1.48E-25	2
	Hsp A2	comp24673_c0_seq1	P54652	88	1.47E-54	1
	Hsp A4	comp12212_c0_seq1	Q61316	91	3.38E-158	1
		comp7589_c0_seq1	P34932	79	<1.00E-180	2
	Hsp 90	comp48547_c0_seq1	P07900	77	7.45E-61	2
		comp5586_c0_seq1	Q4R4P1	76	<1.00E-180	1
		comp32336_c0_seq1	O61998	77	8.80E-33	3
Constitutive heat shock proteins [70,71]	Hsp83	comp28290_c0_seq1	P12861	95	1.42E-43	3
	Hsc20	comp17434_c0_seq1	Q8K3A0	64	3.26E-60	1
	Hsc70	comp29081_c0_seq1	Q9U639	80	3.69E-88	1
	Hsc71	comp7828_c0_seq1	P08108	98	<1.00E-180	4
Small heat shock proteins [70,71]	Hsp β8	comp23233_c0_seq1	Q9UJY1	67	6.80E-66	1
Oxidative stress responses						
Antioxidant systems [72,73]	Catalase	comp3837_c0_seq1	Q9PT92	93	<1.00E-180	1
	(Cu & Zn) superoxide dismutase	comp21322_c0_seq1	Q751L8	61	2.29E-30	1
		comp496_c0_seq1	O73872	87	3.87E-88	2
		comp17555_c0_seq1	P82205	64	2.05E-33	1
		comp28438_c0_seq1	Q9C0N4	46	2.83E-09	1
	(Mn) superoxide dismutase	comp29573_c0_seq1	P41978	57	6.78E-51	1
		comp1897_c0_seq1	P07895	86	5.13E-131	1
	Glutathione peroxidases	comp37533_c0_seq1	Q4AEH3	61	6.22E-27	2
		comp18221_c0_seq1	Q4RSM6	90	5.39E-121	1
		comp559_c0_seq1	Q4AEI2	80	9.44E-87	1
		comp841_c0_seq1	P00435	81	8.33E-86	1
	Thioredoxin and glutathione reductase	comp2236_c0_seq1	Q86VQ6	85	<1.00E-180	1
	Thioredoxin	comp26993_c0_seq1	Q9BDJ3	72	4.09E-16	1
		comp572_c0_seq1	Q9DGI3	90	7.86E-20	1
Methallothioneins [73]	Metal-responsive element-binding transcription factor 2	comp17857_c0_seq1	Q02395	70	3.28E-164	1
Hypoxia induced responses						
Transcription factors [74]	Hypoxia-inducible factor 1α	comp436_c1_seq1	Q9YIB9	76	1.65E-110	1
		comp2126_c0_seq1	Q98SW2	78	<1.00E-180	3
Oxygen transport [74]	Erythropoietin	comp50800_c0_seq1	Q5IGQ0	90	7.05E-47	1
	Hemoglobin β chain	comp12875_c0_seq1	P84652	87	1.01E-78	1
	Myoglobin	comp390_c1_seq1	Q9DGJ1	91	5.58E-72	1
Aerobic/anaerobic metabolism [74]	Malate dehydrogenase	comp11493_c0_seq1	P11708	83	3.41E-37	1
	Succinate dehydrogenase	comp898_c0_seq1	Q7ZVF3	94	<1.00E-180	2
	Citrate synthase	comp44107_c0_seq1	Q91V92	77	6.21E-39	1
	Phosphoglycerate mutase	comp703_c0_seq1	P18669	94	6.82E-163	1
	Phosphoglycerate kinase	comp21645_c0_seq1	Q60HD8	76	2.87E-163	1
	α-enolase	comp324_c0_seq1	Q9PVK2	96	<1.00E-180	1
	Lactate dehydrogenase (A chain, B chain, C chain)	comp4088_c0_seq1	Q92055	99	<1.00E-180	1
		comp192_c0_seq1	P20373	98	<1.00E-180	1
		comp17481_c0_seq1	Q06176	97	7.12E-128	2
	Glycogen phosphorylase	comp28770_c0_seq1	Q9XTL9	79	2.43E-178	3
Metabolic rate suppression [74]	α-tropomyosin	comp1635_c0_seq2	P84335	99	4.56E-56	2
		comp1488_c0_seq1	P13105	96	1.61E-37	1
	Myosin heavy chain	comp37734_c0_seq1	Q63357	87	2.22E-41	1
	Insulin-like growth factor binding protein 1	comp12811_c0_seq1	P24591	55	5.49E-43	1
Sulfide detoxification						
Sulfide metabolism and toxicity [75-77]	Sulfide:quinone oxidoreductase	comp1919_c0_seq1	Q9Y6N5	85	<1.00E-180	1
	Sulfite oxidase	comp25579_c0_seq1	P07850	78	2.59E-93	2
	Sulfur dioxygenase (ETHE1)	comp1681_c0_seq1	Q9DCM0	78	3.54E-104	2
	Thiosulfate sulfurtransferase (Rhodanese)	comp2624_c0_seq1	Q8NFU3	62	2.19E-28	2
		comp12736_c0_seq1	Q3U269	71	1.22E-176	1
	Mercaptopyruvate sulfurtransferase	comp1051_c1_seq7	P97532	74	1.81E-15	1
	Cytochrome c oxidase complex (complex III subunit 6, subunit 3)	comp513_c0_seq3	P07919	84	1.75E-18	1
		comp4_c0_seq1	Q96133	93	3.16E-141	1

For each candidate genes, we reported the database and accession number, percent similarity and E-value, as well as the total number of contigs that matched to a specific database record.

### SNP discovery

We identified SNPs by mapping RNAseq reads back to the reference transcriptome using BWA and calling SNPs using the GATK pipeline [61,62]. Sites were limited to those for which data for all six individuals were available, and 106,524 sites had confident genotype calls across all six individuals. 18,703 transcripts had at least 1 SNP with a median of 4 SNPs and mean of 5.7 SNPs per transcript. In this set, there are 41,777 synonymous and 21,706 non-synonymous mutations in coding sequences. 43,228 SNPs were found in untranslated regions. The transcript with the largest number of SNPs (74 SNPs) was comp1948_c0_seq1 (bromodomain containing 2 protein; 3,679 nt long).

Of the 106,524 SNPs, 1,566 sites are fixed for alternate alleles between the two drainages; 280 of these represented non-synonymous differences in 250 contigs. Sites with SNP calls in all individuals were combined into an excel spreadsheet for accessibility to researchers in the field. This database is available under: http://www.sulfide-life.info/mtobler/databases.

### Differential expression across drainages

**Figure 3:**
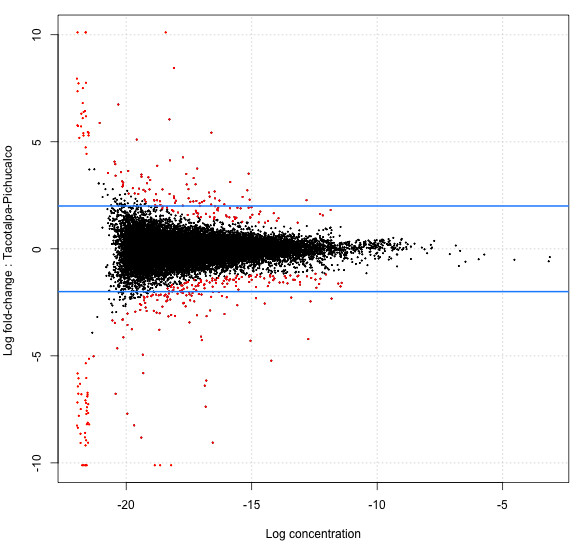
**Plot of the log-fold change between the two drainages versus the log-concentration for each transcript. **The most differentially expressed transcripts (*P* ≤ 0.01) are colored in red. The blue lines are a log-fold change of 2, indicating a fold change of 4.

We compared expression levels in biological replicates across the two drainages investigated to identify loci that are differentially expressed in *P. mexicana* from the two drainages. 21,480 transcripts were analyzed for differential expression. 382 transcripts (representing less than 2% of analyzed transcripts) showed evidence for differential expression (with a *P*-value ≤ 0.01) between the two drainages. 229 transcripts (59.9% of differentially expressed transcripts) were up-regulated in the Pichucalco compared to Tacotalpa drainage, and 153 genes were up-regulated in Tacotalpa compared to Pichucalco drainage (Figure 3).

Of the 382 differentially expressed transcripts, 251 had hits either in the SwissProt or NR databases in our annotation database; 172 of them were also annotated with GO IDs. Overall, 87 sequences were annotated with a molecular function term (these mostly related to binding and catalytic activity), 82 sequences with biological process term (mostly relating to metabolic processes, cellular processes, and biological regulation), and 83 sequences with a cellular component term (mostly cell and organelle; see Additional file 4: Figure S3 for details).

## Discussion

This study used RNA sequencing and *de novo* assembly to characterize and annotate the transcriptome of *Poecilia mexicana*, a livebearing fish used in various evolutionary ecology studies. We identified over 53,000 putatively unique loci, which mapped to over 20,300 unique sequences in the SwissProt and NR databases. Reciprocal blast searches to other published fish transcriptomes indicated that *P. mexicana* transcripts detected in our study provided high concordance (between 51 and 74%) with the guppy, medaka, stickleback, and zebrafish transcriptomes. We also validated six of the predicted transcripts using RT-PCR and sequencing. This provides strong evidence that the contigs we assembled in absence of a reference genome largely represent real transcripts and not assembly error.

To further investigate the congruence of our draft *P. mexicana* transcriptome to that of the most closely related species available, the congeneric guppy, we annotated both transcriptomes with gene ontology (GO) IDs for quantitative comparison. While the GO ID representation uncovered for *P. mexicana* qualitatively matched that of other fish transcriptomes [e.g., [54,78], we found significant deviations in the majority of GO ID representations between *P. mexicana* and the guppy. These differences in GO ID representations across the two closely related species likely stem from differences in methodologies between the studies, specifically the tissue choice and sample preparation. Our approach captured a high proportion of the guppy transcriptome (66% of transcripts identified by blast; only 55% of transcripts were matched in the reciprocal comparison). This is surprising because we focused solely on the gills of females, while the guppy transcriptome was based on a broader sample of tissues (brain and body) from both sexes [54]. This suggests that many loci are transcribed at sufficient levels in gill tissue for transcriptome assembly. Based on comparative analyses with other fish transcriptomes, we achieved a similar (or higher) degree of representation with our approach; we attribute this to our focus on a single sex, a single tissue, and a single ecotype primarily to mitigate potential problems in *de novo* assembly in absence of a reference genome. For example, reciprocal blast searches to model organism transcriptomes consistently revealed higher congruence for *P. mexicana* (this study) than in the guppy [54]. In summary, our approach resulted in high coverage and high congruence with other available fish transcriptomes, which renders the *P. mexicana* transcriptome a useful resource for developing genomic tools in future evolutionary ecology studies.

Among the most peculiar aspects of *P. mexicana*’s biology is its tendency to invade extreme environments in the form of caves and toxic, hydrogen sulfide-rich springs. Populations in extreme environments are characterized by convergent patterns of adaptive trait divergence and ongoing ecological speciation [38,40]. The genomic tools developed here will allow for an increased focus on elucidating the genetic basis of evolution in extreme environments. Annotation of transcripts based on matches to major databases revealed the presence of a diversity of candidate genes relevant to dealing with physiochemical stressors, which will be instrumental for hypothesis testing in upcoming comparative studies between ecotypes. These candidate genes pertain to general stress responses, such as heat shock proteins and oxidative stress responses, that could provide insights about the mechanisms underlying selection against immigrants across ecologically divergent habitat types [46-48]. Candidate genes also include more system-specific pathways pertaining to sulfide metabolism and hypoxia-induced responses. Sulfide spring ecotypes are perpetually exposed to high concentrations of hydrogen sulfide and low oxygen concentrations [79]; hence, analyzing structural changes in candidate proteins and changes in gene regulation across ecotypes residing in sulfidic and non-sulfidic environments should be a high priority.

Sulfide springs have been independently colonized by *P. mexicana* in at least three different drainages [38], and future analyses of ecotype differentiation between sulfidic and non-sulfidic habitats also have to anticipate potential drainage specific effects. We identified over 1,500 putatively fixed alleles between the two drainages, 280 of which were characterized as non-synonymous mutations in coding regions. Our sample size of three individuals per drainage does not have adequate power to call fixed differences with certainty, nor can we draw any conclusions about the adaptive value of different alleles. However, the present analysis corroborates some previously detected geographic structuring across drainages [38] and provides a set of markers for future studies.

Besides allelic variation across drainages, we were also able to document significant variation in gene regulation. We uncovered some noise in the expression patterns quantified, with single individuals having highly up- or down-regulated gene expression for certain loci. Individual expression outliers could be driven by a variety of factors in the sampling procedure; e.g., age, reproductive state, history of parasitization, and other potentially relevant factors, which were not quantified for individual specimens, could all affect transcription of particular genes. Nonetheless, the expression analysis allowed us to identify over 350 transcripts that were consistently up- or downregulated between the drainages. These differences in gene regulation may be due to genetic divergence among populations of different drainages, or they may reflect plastic differences in gene expression in response to large-scale environmental factors. Clearly, uncovering the proximate and ultimate mechanisms underlying differential gene regulation need to be explored in future studies.

## Conclusion

The newly sequenced, assembled, and annotated transcriptome of *P. mexicana* provides a valuable genomic resource to study the molecular underpinnings of adaptation to extreme environment in replicated sulfide spring and cave environments. This dataset also contributes to the growing number of genomic resources available for species of the family Poeciliidae [e.g., [54,80-86], which are broadly studied as model organisms for behavior, ecology, evolution, and medical genetics [87]. Together, these newly developed genomic tools provide valuable resource for ecological genomics projects, since we can build upon an extensive collection of data on phenotypic variation and the evolutionary forces shaping this variation across populations and species in this family (see [87,88] for broad overviews).

## Availability and requirements

Data files with raw sequence information were deposited in the Sequence Read Archive on Genbank (Study Accession ID: SRA056996). The transcriptome, annotation summaries, and SNP data is publicly available at http://www.sulfide-life.info/mtobler/databases.

## Competing interests

The authors declare that they have no competing interests.

## Authors’ contributions

JLK, MP, and MT conceived of the study and participated in design and implementation of the study. LRA and MT conducted fieldwork. MCY and JLK conducted RNA-seq sample and library preparation. JLK, CNP, MT performed all computational and statistical analysis, and JLK and MT prepared the manuscript. All authors read and approved the final manuscript.

## Supplementary Material

Additional file 1: Figure S1 Map of the study locations with the three major towns in the area for orientation. Site (1) represents Arroyo Rosita in the Río Pichucalco drainage; site (2) Arroyo Bonita in the Río Tacotalpa drainage. The insert depicts the location of the study area (black square) in Mexico. Click here for file

Additional file 2: Table S1 Transcripts for RT-PCR validation and corresponding primers. Click here for file

Additional file 3: Figure S2 Phylogenetic relationships between species used in the comparative transcriptome analysis after Li *et al*. [89]. Click here for file

Additional file 4: Figure S3 Blast2GO assignment for 172 annotated sequences that were differentially expressed between *P. mexicana* from the Tacotalpa and Pichucalco river drainages. The numbers next to each colored slice of the pie chart represent the number of genes in the respective category. Click here for file
