# Supplementary figures and images for: Genomic resources for a model in adaptation and speciation research: characterization of the Poecilia mexicana transcriptome
Source: BMC Genomics. 2012 Nov 21;13:652. doi: 10.1186/1471-2164-13-652 (PMC3585874; doi:10.1186/1471-2164-13-652)

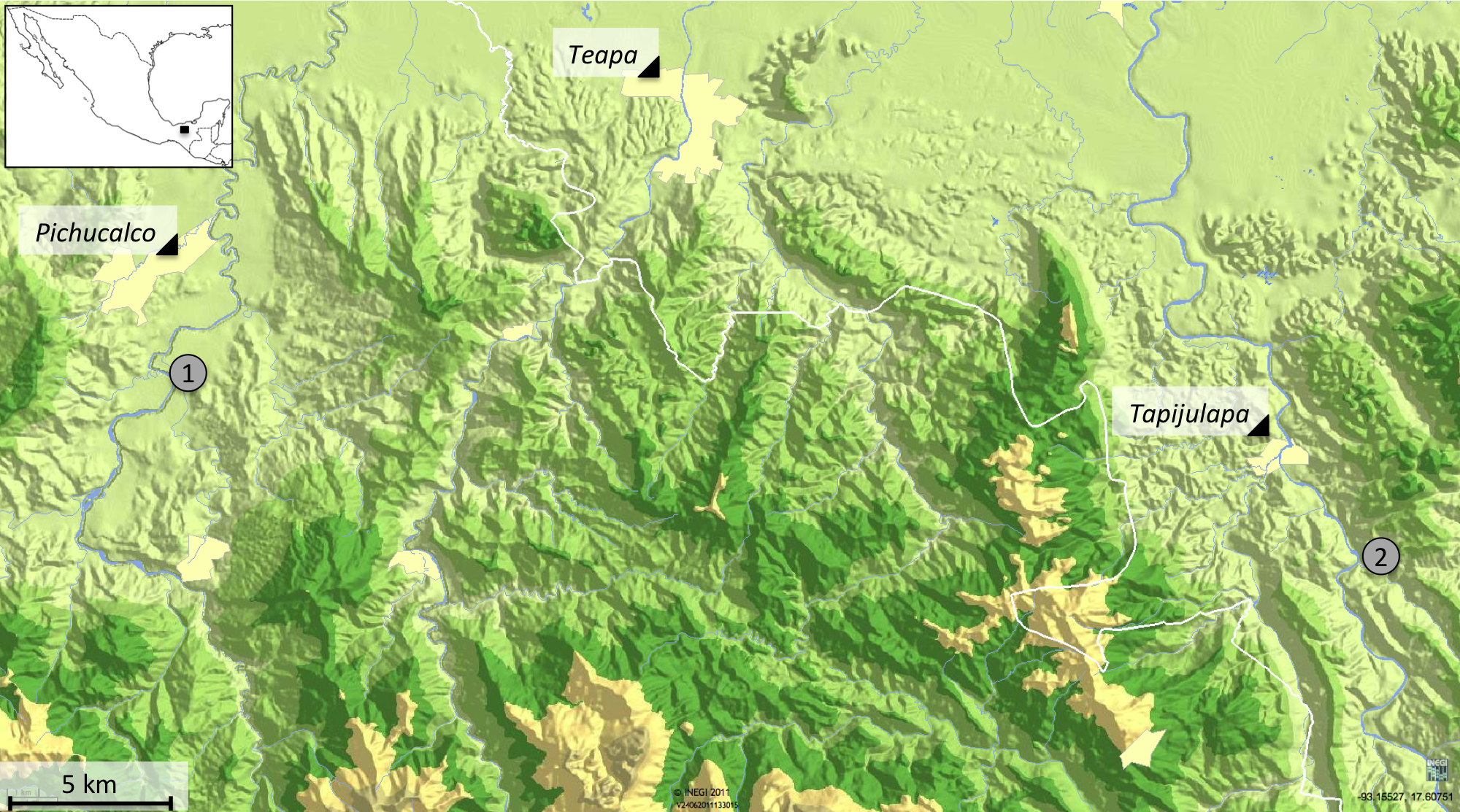

Supplement: Additional file 1: Figure S1 — Map of the study locations with the three major towns in the area for orientation. Site (1) represents Arroyo Rosita in the Río Pichucalco drainage; site (2) Arroyo Bonita in the Río Tacotalpa drainage. The insert depicts the location of the study area (black square) in Mexico. [file 1471-2164-13-652-S1.tiff]

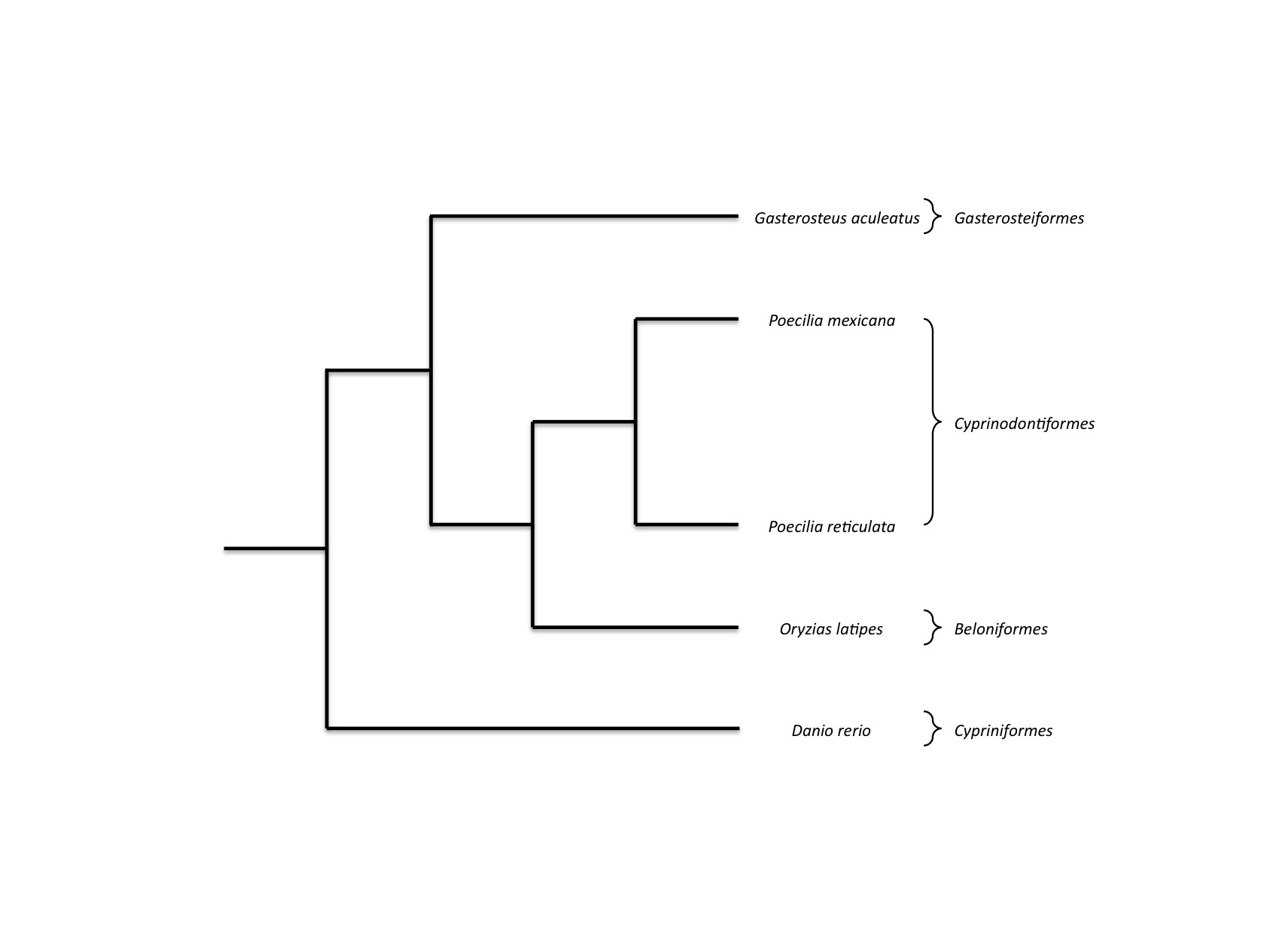

Supplement: Additional file 3: Figure S2 — Phylogenetic relationships between species used in the comparative transcriptome analysis after Li et al. [89]. [file 1471-2164-13-652-S3.jpeg]

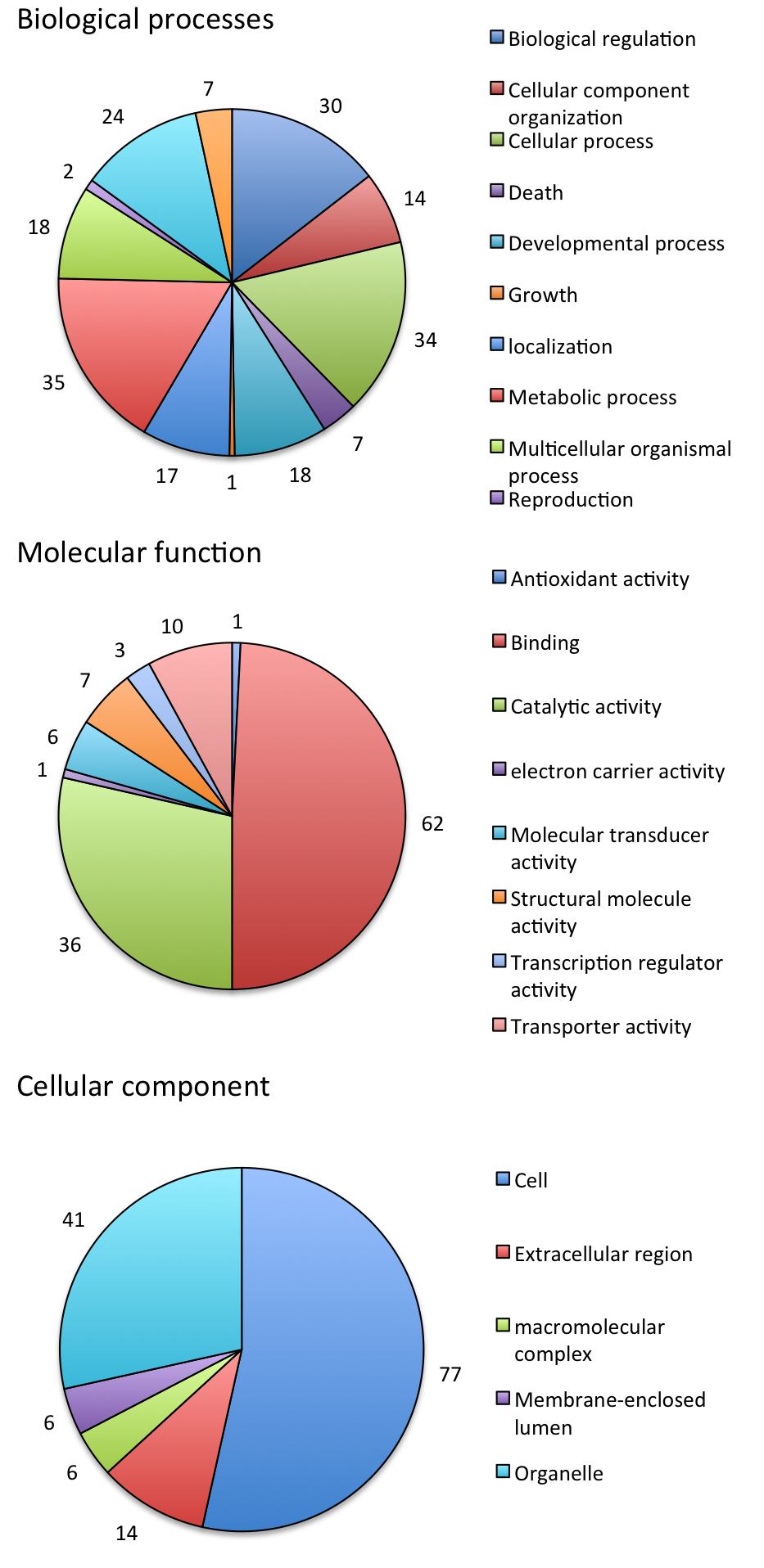

Supplement: Additional file 4: Figure S3 — Blast2GO assignment for 172 annotated sequences that were differentially expressed between P. mexicana from the Tacotalpa and Pichucalco river drainages. The numbers next to each colored slice of the pie chart represent the number of genes in the respective category. [file 1471-2164-13-652-S4.tiff]
